# Effect of gabapentin on solution surface properties and micellization behavior of betaine-based surfactant ionic liquids

**DOI:** 10.1038/s41598-024-83777-7

**Published:** 2025-01-02

**Authors:** Shima Ghasemzadeh, Mohammad Bagheri, Hemayat Shekaari, Behrang Golmohammadi

**Affiliations:** Department of Physical Chemistry, University of Tabriz, Tabriz, Iran

**Keywords:** Surface tension, Electrical conductivity, Betaine, Ionic liquids, COSMO, Green chemistry, Medicinal chemistry, Surface chemistry, Theoretical chemistry

## Abstract

This study investigates the surface properties, micellization, and electrical conductivity of betaine-based ionic liquids (ILs) composed of [R-bet][Br] where R represents the C_4_, C_6_ and C_8_ (specifically [C_4_bet][Br], [C_6_bet][Br], and [C_8_bet][Br]) in aqueous gabapentin solutions at concentrations of (0.0000, 0.0100, 0.0300, and 0.0500) mol kg^−1^ at 298.15 K. The surface tension measurements revealed that increasing gabapentin concentration and alkyl chain length decrease surface tension, indicating significant hydrophobic and hydrophilic interactions. The related thermophysical micellization parameters, including critical micelle concentration (CMC) and minimum surface area per molecule (*A*_*min*_), exhibited improved micellization and interfacial efficiency with longer alkyl chains. Thermodynamic analysis confirm the spontaneous nature of micelle formation, with more negative Gibbs free energy values for SAILs with longer alkyl chains. The electrical conductivity studies indicate lower limiting molar conductivity (*Λ*_0_) at higher gabapentin concentrations, due to increased viscosity and ion-ion interactions. Ion association constants (*K*_*A*_) and DFT-COSMO calculations support stronger hydrophobic interactions and molecular packing influenced by alkyl chain length and gabapentin.

**Supplementary Information:**

The online version contains supplementary material available at 10.1038/s41598-024-83777-7.

## Introduction

Betaine is known as a neutral chemical compound bearing a positively charged cationic functional group, devoid of a hydrogen atom. This is typically exemplified by a quaternary ammonium or phosphonium cation, alongside a negatively charged functional group, such as a non-adjacent carboxylate group. Known by various names including betaine anhydrous and trimethyl glycine (TMG). It is a naturally founded substance in the human body. Betaine plays a crucial role in liver function, cellular reproduction, and in the synthesis of carnitine. Notably, it facilitates the conversion of the amino acid homocysteine into methionine. With its wide availability, cost-effectiveness, and biocompatibility, surface-active properties, betaine holds promise for future applications in pharmaceutical industrial^1–4^.

Gabapentin is an antiepileptic, medication that has been utilized for many purposes such as treating neuropathic and spasmatic pain. It is increasingly prescribed off-label for alcohol withdrawal, chronic pain, and drug use disorders. Its use has risen significantly, but concerns about abuse, particularly among opioid-dependent patients, are growing. The drug has been often co-prescribed with opioids, and many patients in chronic pain clinics. Those prescribed gabapentin are more likely to fill opioid prescriptions and have co-occurring substance use or mood disorders^5–12^.

Gene therapy and experimental treatments for opioid addiction are areas of ongoing research, with the goal of developing more effective and long-lasting treatments for individuals struggling with opioid use disorder (OUD). These approaches aim to address the underlying biological and neurological mechanisms of addiction, which are not adequately targeted by current therapies. While betaine alkyl ester bromides are not directly used in treating opioid addiction, their role in drug delivery systems could theoretically support the administration of gene therapies or other novel treatments. These advancements could potentially offer new hope for individuals struggling with opioid use disorder, providing more effective and lasting solutions compared to current treatment options^13–17^.

The most important point for gene therapy treatment for opioid persons addicted to gabapentin, where alkyl betaine ester bromide (ABEB ILs) are used in the components of the therapy sessions, is the potential for ABEB ILs to enhance the delivery and efficacy of gene therapy vectors. These unique compounds can improve cellular uptake, intracellular trafficking, and reduce immunogenicity, leading to more effective and targeted therapies. Surface tension and conductance measurements can provide valuable insights into the interactions between ABEB ILs and gene therapy vectors. Changes in these properties can indicate the strength of vector-ABEB IL interactions, cellular membrane interactions, and ion transport, all of which are important factors in gene therapy delivery and efficiency^18,19^.

Ionic liquids (ILs) and their binary mixtures with co-solvents exhibit unique physicochemical properties suitable for diverse applications. Mixtures of 1-butyl-3-methylimidazolium bis(trifluorosulfonyl)imide ([bmim][Tf_2_N]) with polyethylene glycols (PEGs) demonstrate significant synergistic solvation effects, particularly with PEG-600, attributed to hydrogen bonding and dipolar interactions, as evidenced by solvatochromic probe studies^20^. Cationic gemini surfactants, such as hexanediyl-1,6-bis(dimethylcetylammonium bromide) (16-6-16), show increased critical micelle concentration (CMC) and reduced stability in the presence of adenosine, highlighting the impact of additives and temperature on micellization behavior^21^. Similarly, the addition of hydrotropes like 3-nitrobenzene sulfonic acid sodium salt (NBS) to sodium dodecyl sulfate (SDS) reduces the CMC, indicating synergistic effects and improved micellar properties^22^. Ionic liquids such as 1-ethyl-3-methylimidazolium hexafluorophosphate ([emim][PF_6_]) modify SDS micellar behavior, reducing CMC and altering aggregation dynamics. Longer alkyl chains in the IL cation further enhance these effects, demonstrating the potential for tailored micellar systems^23^. Thermophysical studies of protic and aprotic polar solvent mixtures reveal non-ideal behavior, with mixtures of water and aprotic solvents displaying improved thermal stability and dynamic viscosity profiles^24^. The mixed micellization of amitriptyline hydrochloride (AMT) with poly(ethylene glycol) t-octylphenyl ether (TX-100) shows decreased CMC and favorable thermodynamic properties, suggesting optimized interactions in drug-surfactant systems^25^. Finally, micellization of pentanediyl-1,5-bis(dimethylcetylammonium bromide) (16-5-16) in the presence of asparagine shows reduced stability with increasing amino acid concentrations and temperatures, emphasizing the importance of molecular interactions in surfactant systems^26^. These studies provide valuable insights into the molecular-level behavior of ionic liquid, surfactant, and solvent systems for industrial and technological applications.

Surface-active ionic liquids (SAILs) present a promising approach to addressing this challenge. Their unique amphiphilic nature, characterized by the coexistence of hydrophilic and lipophilic moieties within the same molecule, coupled with their structural versatility, offers significant potential for enhancing drug permeability, formulation, and delivery. Betaine-based ionic liquids, a specific subclass of SAILs, combine the properties of ionic liquids and betaines, exhibiting tunability and surface activity, making them particularly promising for pharmaceutical applications such as improving drug solubility and permeability^27–29^. The ionic nature of betaine-based ILs, arising from the presence of charged species, coupled with their environmentally friendly characteristics, positions them as desirable candidates for enhancing drug properties^30–32^. The tunability of betaine-based ILs allows for the precise tailoring of their properties to meet specific pharmaceutical requirements, further enhancing their potential for drug delivery applications^33,34^. To delve deeper into the intricate interactions between SAILs in aqueous gabapentin solution, this study employs surface tension and electrical conductivity measurements. These techniques offer a window into the fundamental principles governing micellization and electrical conductivity, shedding light on how drugs navigate the complexities of solutions and engage with other molecules^35–37^. Micellization, the elegant process of surfactants assembling into micelles, presents a promising avenue for enhancing drug permeability and bioavailability.

Electrical conductivity and static surface tension measurements are generally considered the most reliable methods. These techniques can provide valuable insights into ion mobility and interfacial behavior influenced by micelle formation. The surface tension and electrical conductivity measurements were employed at 298.15 K and ambient pressure to determine the critical micelle concentration (CMC) and limiting molar conductivity (*Λ*_0_) of the SAILs in the presence of aqueous gabapentin solutions. Additionally, COSMO analysis was conducted to gain a more in-depth understanding of the intermolecular interactions between gabapentin and SAILs molecules in aqueous media. To measure surface tension and electrical conductivity, several aqueous gabapentin solutions containing varying concentrations of SAILs were prepared. A static force tensiometer equipped with the Wilhelmy plate method was employed to obtain the surface tension data. Out of the various utilized methods for measuring the surface tension the Wilhelmy plate methods offers the highest accuracy and reliability. These measurements allowed for the calculation of surface-related properties, including the CMC, Gibbs free energy of micellization $\Delta {G_{mic}}$, and minimum surface area per surfactant molecule *A*_min_.

Interfacial electron density, another approach to the surface characteristics of a molecule, can be achieved through density functional theory (DFT) calculations. A simple and practical DFT calculation provided by Dmol3, known as the conductor-like screening model (COSMO), offers the surface and total area of cavity *A* and cavity volume *V*, dielectric solvation energy, amount of highest occupied molecular orbital (HOMO), amount of lowest unoccupied molecular orbital (LUMO) and their related *σ*-profile as dielectric characteristics of the chemical structure. This approach can provide DFT-based properties that can help interpret the observed macroscopic results from a microscopic perspective. The *σ*-profile of a molecule provides substantial information about the electrostatic distribution on the molecule structure. Therefore, DFT calculations can offer another microscopic approach to the phenomenological aspect of CMC and molecular structure of ILs in the aqueous gabapentin solutions.

The effect of ionic liquids 2-butoxy-N,N,N-trimethyl-2-oxoethan-1-amonium bromide (betaine butyl ester bromide, [C_4_bet][Br]), 2-hexoxy-N,N,N-trimethyl-2-oxoethan-1-amonium bromide (betaine hexyl ester bromide, [C_6_bet][Br]), and 2-octoxy-N,N,N-trimethyl-2-oxoethan-1-amonium bromide (betaine octyl ester bromide, [C_8_bet][Br]) on the surface behavior of the aqueous gabapentin solutions have been studied. Accordingly, the surface tension and eletrical conductance of the aqueous solutions of these ionic liquids in the presence of gabapentin have been measured and corresponding themodynamic properties of the micellization have been obtained. Also, the DFT calculation has been performed and the cavity surface and volume has been obtained from the COSMO calculation to achieve a comprehensive perspective to the micellization behavior of the ionic liquids.

## Materials and methods

### Chemicals

Table 1 outlines the specifications of the chemicals utilized in this study, including their chemical name, source, molar mass, purity and the CAS.no. The deionized-double distilled water was used in this research with the specific conductance of less than 1 µS·cm^−1^.

**Table 1:** Description of the utilized materials sample.

Chemical name	Source	Molar mass	Purity	CAS.no
Gabapentin	Merck	171.24	≥ 99.9	60142-96-3
Betaine	Merck	117.15	≥ 98	107-43-7
Acetonitrile	Merck	41.05	≥ 99.9	75-05-8
Diethyl ether	Merck	74.12	≥ 99.7	60-29-7
1-bromobutane	Merck	137.02	≥ 99.9	109-65-9
1-bromohexane	Merck	165.07	≥ 99	111-25-1
1-bromooctane	Merck	193.13	≥ 99	111-83-1
Betaine butyl ester bromide [C_4_bet][Br]	Synthesized	254.17	≥ 99	
Betaine hexyl ester bromide [C_6_bet][Br]	Synthesized	282.22	≥ 99	
Betaine octyl ester bromide [C_8_bet][Br]	Synthesized	310.28	≥ 98	

### Synthesis process of the SAILs

**Fig. 1:**
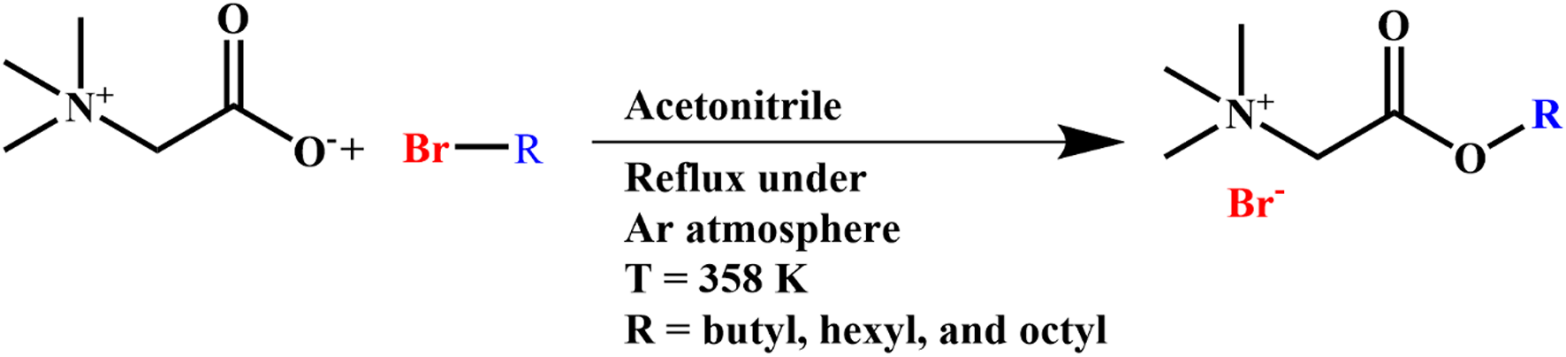
The reaction between betaine and alkyl halid for preparation of Betaine alkyl ester bromide.

Betaine (1 mol), alkyl halide (1.2 mol), and 50 mL of acetonitrile were mixed in a flask and stirred at 358 K for 72 h under reflux and an argon atmosphere. The schematic of the reaction are given in Fig. 1.

The solvent and excess alkyl halide were subsequently removed through distillation and vacuum distillation until a the cream color powder was obtained. Diethyl ether was used to wash and remove residue of the impurities such as unreacted alkyl bromide. The solid precipitates were dried under vacuum to yield the final product. The synthesized ionic liquids were characterized by ^1^H-NMR and FT-IR spectroscopy and these results are included in Figs. S1–S4 in supporting information. Karl–Fisher analysis was also employed to determine the water content of the synthesized ILs. These information have been included in the supporting information of this manuscript and the ILs show purity of higher than 97% with a yield of more than 94%.

### Measurements of surface tension and electrical conductivity

The varied concentration of the stock solutions of ILs in aqueous gabapentin solutions have been prepared through utilization of the analytical balance AND, GR220 with the precision of 1 ± 10^−8^ kg and it was calibrated before using. The specific conductance of ILs electrolytes in the presence of various concentrations (0.0000, 0.0100, 0.0300, and 0.0500 mol·kg^−1^) of gabapentin was measured at 298.15 K. A digital electrical conductometer (Metrohm model 712, Switzerland) equipped with a platinized electrode dipping conductivity cell (cell constant of 0.867 cm^−1^) was used to measure conductance. The conductivity cell was calibrated with a 0.01 mol·kg^−1^ KCl solution as standard. The studied solutions were maintained at a constant temperature using a thermostatic bath. The conductivity electrode was immersed in precisely measured stock solutions of ILs in aqueous gabapentin solution, and the cell was tightly sealed. A thermostatically controlled bath (Julabo ED Germany) was used to circulate water around the double-walled cell, ensuring a temperature precision of 0.02 K. The CMC point of ILs in aqueous gabapentin solutions was determined by extrapolating the inflection point in the specific conductance *κ*, graph of ILs versus their respective concentration^38–41^.

The surface tension measurements were conducted using a Krüss K20 Easy Dyne static tensiometer equipped with a Wilhelmy plate (PL22 provided by the company). The static surface tension instrument presented a microbalance resolution of 10 µg, for surface tension measurements of ILs in varying concentrations of aqueous gabapentin solutions at a constant temperature of 298.15 K. A resistive platinum thermometer immersed in the sample container measured the temperature. The combined standard uncertainties for surface tension data were calculated considering temperature variations, tensiometer device accuracy, and microbalance accuracy. The surface tension measurement accuracy was estimated to be ± 0.1 mN·m^−1^. Accurate data required meticulous cleaning of the Wilhelmy plate between each measurement, involving rinsing with ultrapure, double-distilled, deionized water, followed by high-purity acetone, and finally heating to a red-hot state^42,43^. The critical micellization concentration (CMC) was determined by extrapolating the inflection point observed in the surface tension versus molality plot. This graphical method, based on the intersection of pre- and post-micellization regimes, provides a robust approach for CMC determination and facilitates the calculation of thermodynamic properties like Gibbs free energy^44,45^.

The measurement methods involve various potential errors and uncertainty sources. For electrical conductivity measurements, calibration of the electrical conductometer using a KCl solution may introduce errors if the standard solution is not accurately prepared, and temperature variations, even with thermostatic control (± 0.02 K), can affect ion mobility. The condition of the platinized electrode, device accuracy, and solution preparation may further contribute to systematic and random uncertainties. For surface tension measurements, calibration and cleaning of the Wilhelmy plate are critical, as improper cleaning can leave residues that affect accuracy. Temperature variations measured by a resistive platinum thermometer (± 0.02 K precision) and the microbalance resolution (± 10 µg) are additional sources of error. Graphical determination of the critical micelle concentration (CMC) adds subjective uncertainty due to the interpretation of inflection points. Environmental factors such as air pressure and humidity, along with human handling during sample preparation, also play a role in measurement variability. To ensure accuracy, rigorous calibration, effective cleaning protocols, and consistent experimental conditions are essential.

### DFT calculations

The DFT calculation including the geometery optimization followed by the energy optimization beside the hydration energy calculations have been carried out in the Dmol3 modoul of the Materials studio software. The utilized functional was GGA, VWN-BP and the DND 3.5 basis set has been selected and the water was used as dielectric media for solvation properties.

## Results and discussion

### Surface tension results

**Fig. 2:**
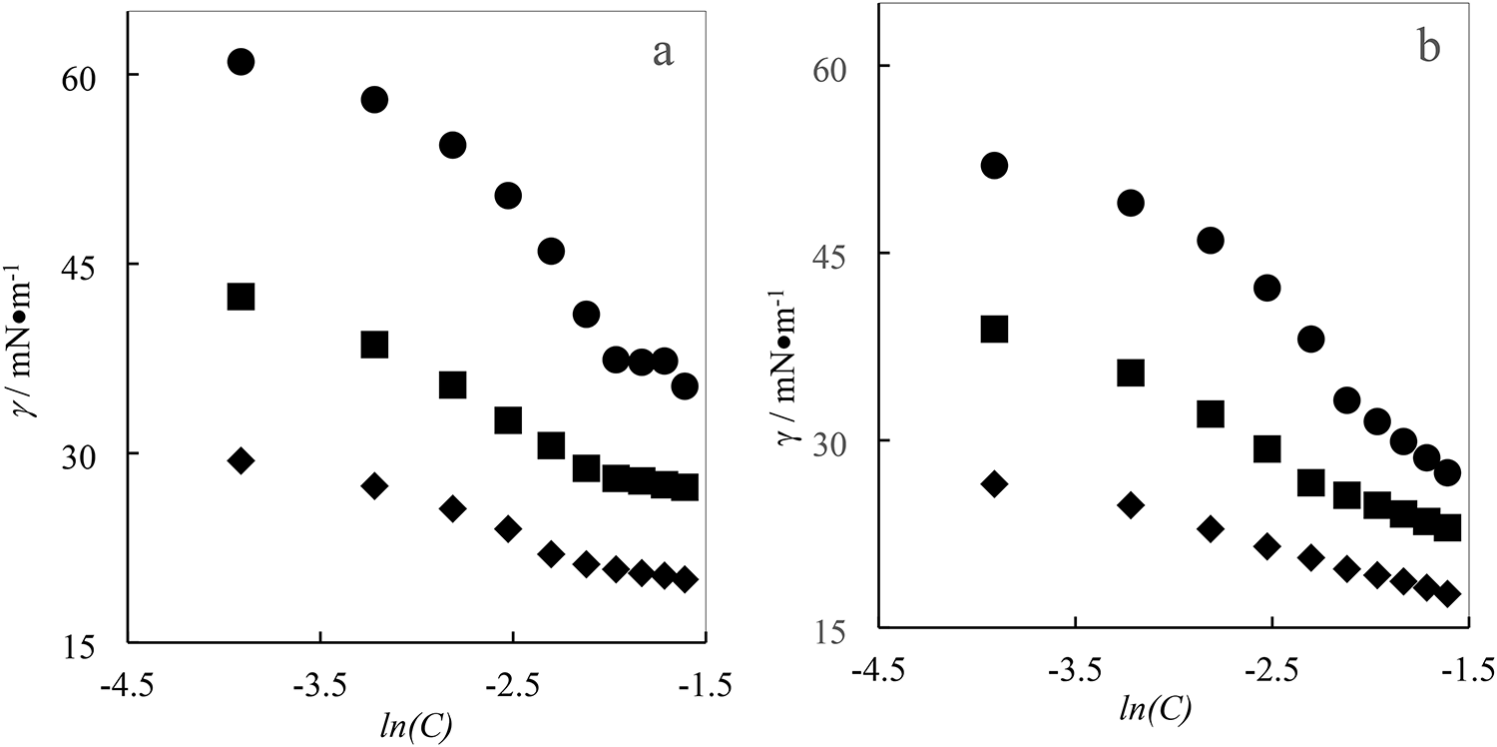
Surface tension (γ) of betaine-based ILs: (**a**) ([C_4_bet][Br] (●), [C_6_bet][Br] (■), and [C_8_bet][Br] (♦)) in aqueous solutions and (**b**) ([C_4_bet][Br] (●), [C_6_bet][Br] (■), and [C_8_bet][Br] (♦)) in aqueous solutions of 0.01 mol kg^−1^gabapentin.

The ILs surface tension (*γ*) in the presence of aqueous gabapentin solutions with varying concentration range (0.0000, 0.0100, 0.0300 and 0.0500 mol·kg^−1^) have been investigated at 298.15 K. The experimental data have been presented in Table 2. Also, these results have been illustrated in Fig. 2.

**Table 2:** Surface tension of betaine based ILs in various aqueous gabapentin solutions (from 0.00 to 0.05 ($mol \cdot k{g^{ - 1}}$) at 298.15 K.

m_GPT_/mol kg^−1^							
0.00		0.01		0.0299		0.0499	
*C/* mol kg^−1^	*γ*/mN m^−1^	*C/* mol kg^−1^	*γ*/mN m^−1^	*C/* mol kg^−1^	*γ*/mN m^−1^	*C/* mol kg^−1^	*γ*/mN m^−1^
[C_4_bet][Br]							
0.0200	61.0	0.0201	52.0	0.0200	45.3	0.0200	38.0
0.0400	58.1	0.0401	49.1	0.0400	42.8	0.0401	35.3
0.0600	54.4	0.0601	46.3	0.0601	39.7	0.0600	31.1
0.0800	50.4	0.0801	42.2	0.0799	35.1	0.0797	25.4
0.0999	46.0	0.1000	38.1	0.1004	28.5	0.0999	22.5
0.1201	41.0	0.1202	33.2	0.1200	26.2	0.1199	20.4
0.1400	37.4	0.1403	31.5	0.1401	24.6	0.1411	18.3
0.1599	37.2	0.1603	29.9	0.1599	22.5	0.1589	16.7
0.1799	37.3	0.1803	28.6	0.1800	20.2	0.1790	13.6
0.2007	35.3	0.2006	27.4	0.1982	18.3	0.1995	12.4
[C_6_bet][Br]							
0.0200	42.4	0.0200	38.9	0.0200	33.7	0.0201	30.1
0.0399	38.6	0.0400	35.4	0.0399	30.5	0.0400	25.4
0.0599	35.4	0.0598	32.1	0.0599	27.0	0.0599	21.6
0.0796	32.6	0.0798	29.3	0.0800	23.6	0.0794	19.4
0.0999	30.6	0.0998	26.6	0.0997	22.1	0.1001	17.6
0.1196	28.8	0.1199	25.6	0.1200	20.8	0.1199	16.4
0.1380	28.0	0.1397	24.8	0.1399	19.5	0.1392	14.1
0.1602	27.8	0.1576	24.1	0.1595	18.4	0.1599	13.8
0.1801	27.5	0.1782	23.5	0.1795	17.6	0.1797	12.8
0.1994	27.3	0.1996	23.0	0.1986	16.9	0.1968	12.0
[C_8_bet][Br]							
0.0200	29.4	0.0200	26.5	0.0200	23.7	0.0200	21.5
0.0399	27.4	0.0400	24.8	0.0400	22.0	0.0399	20.3
0.0600	25.6	0.0598	22.9	0.0599	20.3	0.0593	18.7
0.0800	24.0	0.0800	21.5	0.0800	19.3	0.0801	16.7
0.1000	22.0	0.1000	20.6	0.0998	18.5	0.0999	15.4
0.1190	21.2	0.1198	19.7	0.1199	18.0	0.1195	14.5
0.1399	20.8	0.1398	19.2	0.1399	17.5	0.1389	14
0.1599	20.5	0.1599	18.7	0.1599	17.3	0.1590	13.4
0.1793	20.3	0.1796	18.2	0.1793	17.0	0.1799	13.0
0.1997	20.0	0.1999	17.7	0.1993	16.3	0.1994	12.4

^a^The standard uncertainties for molality, temperature and pressure were *u* (*C*) = 0.001 mol m^−3^, *u* (*T*) = 0.5 K, and u(*P*) = 0.01 MPa respectively with level of confidence 0.95. The standard combined uncertainty for surface tension were about, *uc* (*γ*) = 0.01 mN·m^−1^ (level of confidence 0.68), respectively.

Through the obtained *γ* data the surface related parameters such as $\Pi$(interface surface pressure), ${\gamma _{CMC}}$ (CMC point surface tension), ${A_{\hbox{min} }}$ (minimum surface area occupied per molecule), ${\Gamma _{\hbox{max} }}$ (Gibbs maximum excess surface concentration) were computed and have been also presented in the Table 3. Through a careful inspection of the Table 3, it was revealed that the values of CMC point and *γ* for the investigated ILs in the presence of varying concentrations of aqueous gabapentin solution show a decreasing trend. The $\Pi$ is an indicator of solvent and solution surface tension difference or in other words illustrates the effect of ILs in lowering the pure solvent surface tension and can be calculated through following formula^46,47^:

1 $$\Pi ={\gamma _0} - \gamma$$

Where *γ*_*0*_, and *γ* illustrate the surface tension of pure solvent and the solution. The Gibbs maximum excess surface concentration ${\Gamma _{\hbox{max} }}$, also can be defined as follows^46,47^:

2 $${\Gamma _{\hbox{max} }}= - \frac{1}{{nRT}}\left[ {\frac{{\partial \gamma }}{{\partial \ln C}}} \right]$$

Where *n*, is the number of ionic species in the studied systems, *R* is the gas constant, *T* is the measured temperature, *γ* is the surface tension of the solutions and *C* represent ILs concentrations in aqueous gabapentin solution. The related values of ${\Gamma _{\hbox{max} }}$ have been tabulated in Table 3.

**Table 3:** Surface active parameters of [C_4_bet][Br] in various aqueous gabapentin solutions (from 0.00 to 0.05 mol·kg^−1^) at 298.15 K.

${\Pi}$	${10^3} \times {\Gamma _{\hbox{max} }}$	${A_{\hbox{min} }}$	$\Delta {G_{mic}}$	$\Delta G_{{ads}}^{^\circ }$	$G_{{\hbox{min} }}^{S}$	$CMC$	${\gamma _{CMC}}$
mN m^−1^	mol m^−2^	Å^2^	kJ mol^−1^	kJ mol^−1^	kJ mol^−1^	mol kg^−1^	mN m^−1^
[C_4_bet][Br] + water							
11.0	1.76	0.094	−19.656	−13.406	2.125	0.15	37.4
14.0	0.932	0.178	−17.934	−2.912	4.013		
17.6	2.551	0.065	−16.932	−10.033	1.466		
21.6	3.798	0.044	−16.219	−10.532	0.985		
26.0	4.315	0.038	−15.669	−9.644	0.867		
31.0	4.144	0.040	−15.216	−7.735	0.903		
34.6	3.387	0.049	−14.836	−4.619	1.104		
34.8	2.142	0.078	−14.508	1.740	1.746		
34.7	0.476	0.349	−14.216	58.639	7.852		
36.7	−1.613	−0.103	−13.945	−36.691	−2.318		
[C_4_bet][Br] in 0.01 mol·kg^−1^ concentration of aqueous gabapentin solution							
20.0	4.932	0.034	−19.651	−15.595	0.758	0.12	37.4
23.0	0.058	2.843	−17.934	375.906	64.042		
26.0	2.316	0.072	−16.933	−5.708	1.615		
29.8	3.551	0.047	−16.222	−7.829	1.053		
33.9	3.815	0.044	−15.674	−6.788	0.980		
38.8	3.518	0.047	−15.218	−4.190	1.063		
40.5	2.948	0.056	−14.835	−1.098	1.269		
42.1	2.282	0.073	−14.506	3.946	1.639		
43.4	1.625	0.102	−14.215	12.496	2.302		
44.6	1.041	0.160	−13.952	28.910	3.594		
[C_4_bet][Br] in 0.0299 mol·kg^−1^ concentration of aqueous gabapentin solution							
27.0	10.400	0.016	−19.669	−17.173	0.262	0.10	28.33
29.2	−0.789	−0.210	−17.950	−51.694	−3.274		
32.3	3.359	0.049	−16.945	−7.421	0.835		
37.0	4.505	0.037	−16.237	−8.060	0.626		
43.5	3.949	0.042	−15.674	−4.555	0.724		
45.8	3.043	0.055	−15.232	0.0025	0.942		
47.4	2.540	0.065	−14.850	3.818	1.116		
49.5	2.813	0.059	−14.523	2.525	0.976		
52.0	4.027	0.041	−14.230	−2.100	0.661		
54.0	6.240	0.027	−13.992	−5.634	0.439		
[C_4_bet][Br] in 0.0499 mol·kg^−1^ concentration of aqueous gabapentin solution							
34.0	−1.783	−0.093	−19.681	−38.755	−1.413	0.08	25.18
36.7	2.408	0.069	−17.957	−2.719	1.045		
41.0	2.998	0.055	−16.958	−3.282	0.84		
47.0	2.891	0.057	−16.255	0.002	0.871		
49.5	2.730	0.061	−15.696	2.435	0.922		
51.6	2.692	0.062	−15.244	3.922	0.935		
53.7	2.823	0.059	−14.843	4.179	0.892		
55.3	3.125	0.053	−14.548	3.145	0.806		
58.4	3.587	0.046	−14.254	2.025	0.702		
59.6	4.193	0.040	−13.985	0.227	0.600		
[C_6_bet][Br] + water							
29.6	0.652	0.255	−19.652	25.768	4.695	0.10	30.60
33.4	1.451	0.114	−17.943	5.077	2.109		
36.6	1.789	0.093	−16.935	3.527	1.711		
39.4	1.824	0.091	−16.231	5.371	1.678		
41.4	1.652	0.101	−15.669	9.396	1.853		
43.2	1.343	0.124	−15.226	16.933	2.278		
44.0	0.959	0.173	−14.871	30.996	3.190		
44.2	0.408	0.407	−14.503	93.751	7.495		
44.5	−0.151	−1.099	−14.213	−308.612	−20.244		
44.7	−0.739	−0.225	−13.961	−74.466	−4.142		
[C_6_bet][Br] in 0.01 mol·kg^−1^ concentration of aqueous gabapentin solution							
33.1	1.709	0.097	−19.659	−0.287	1.543	0.11	25.90
36.6	0.725	0.229	−17.941	32.529	3.636		
39.9	1.293	0.128	−16.947	13.901	2.038		
42.7	1.108	0.150	−16.231	22.293	2.379		
45.4	0.481	0.345	−15.679	78.774	5.485		
46.4	−0.257	−0.645	−15.224	−195.42	−10.239		
47.2	−0.870	−0.191	−14.846	−69.082	−3.029		
47.9	−1.224	−0.136	−14.548	−53.667	−2.153		
48.5	−1.333	−0.125	−14.245	−50.62	−1.977		
49.0	−1.068	−0.156	−13.964	−59.859	−2.469		
[C_6_bet][Br] in 0.0299 mol·kg^−1^ concentration of aqueous gabapentin solution							
38.3	0.042	3.934	−19.673	887.634	55.907	0.08	23.18
41.5	2.041	0.081	−17.959	2.371	1.156		
45.0	2.582	0.064	−16.955	0.470	0.914		
48.4	2.713	0.061	−16.236	1.603	0.870		
49.9	2.758	0.060	−15.693	2.400	0.856		
51.2	2.837	0.059	−15.235	2.812	0.832		
52.5	2.991	0.056	−14.856	2.697	0.789		
53.6	3.231	0.051	−14.53	2.058	0.730		
54.4	3.569	0.047	−14.239	1.006	0.661		
55.1	3.974	0.042	−13.989	−0.126	0.594		
[C_6_bet] [Br] in 0.0499 mol·kg^−1^ concentration of aqueous gabapentin solution							
41.9	−0.055	−3.002	−19.666	−777.061	−25.265	0.07	21.08
46.6	2.229	0.075	−17.966	2.942	0.627		
50.4	2.095	0.079	−16.963	7.088	0.667		
52.6	2.132	0.078	−16.267	8.403	0.656		
54.4	2.374	0.070	−15.696	7.217	0.589		
55.6	2.644	0.063	−15.248	5.779	0.529		
57.9	2.828	0.059	−14.878	5.592	0.494		
58.2	2.863	0.058	−14.537	5.791	0.488		
59.2	2.696	0.062	−14.247	7.715	0.519		
60.0	2.377	0.070	−14.023	11.217	0.588		
[C_8_bet][Br] + water							
42.6	0.184	0.903	−19.655	212.125	11.535	0.12	21.20
44.6	0.797	0.208	−17.940	38.030	2.660		
46.4	1.057	0.157	−16.931	26.976	2.006		
48.0	1.094	0.152	−16.220	27.654	1.938		
50.0	0.988	0.168	−15.669	34.949	2.146		
50.8	0.782	0.212	−15.237	49.735	2.711		
51.2	0.509	0.326	−14.838	85.703	4.163		
51.5	0.187	0.889	−14.506	261.199	11.349		
51.7	−0.176	−0.943	−14.224	−307.876	−12.041		
52.0	−0.583	−0.285	−13.958	−103.195	−3.638		
[C_8_bet][Br] in 0.01 mol·kg^−1^ concentration of aqueous gabapentin solution							
45.5	−0.011	−14.931	−19.658	−4110.643	−185.218	0.10	20.60
47.2	0.987	0.168	−17.943	29.867	2.087		
49.1	1.147	0.145	−16.945	25.859	1.796		
50.5	1.138	0.146	−16.226	28.169	1.811		
51.4	1.107	0.150	−15.673	30.776	1.862		
52.3	1.097	0.151	−15.227	32.46	1.878		
52.8	1.119	0.148	−14.844	32.323	1.840		
53.3	1.177	0.141	−14.512	30.771	1.750		
53.8	1.265	0.131	−14.225	28.292	1.628		
54.3	1.386	0.120	−13.961	25.210	1.486		
[C_8_bet][Br] in 0.0299 mol·kg^−1^ concentration of aqueous gabapentin solution							
48.3	−0.063	−2.641	−19.669	−787.886	−30.697	0.08	19.30
50.0	0.828	0.200	−17.954	42.408	2.330		
51.7	0.823	0.202	−16.952	45.891	2.346		
52.7	0.688	0.241	−16.235	60.345	2.805		
53.5	0.578	0.287	−15.689	76.792	3.336		
54.0	0.529	0.314	−15.234	86.789	3.646		
54.5	0.545	0.305	−14.852	85.113	3.540		
54.7	0.622	0.267	−14.523	73.437	3.104		
55.0	0.753	0.221	−14.24	58.827	2.564		
55.7	0.931	0.178	−13.979	45.845	2.073		
[C_8_bet][Br] in 0.0499 mol·kg^−1^ concentration of aqueous gabapentin solution							
50.5	−0.384	−0.432	−19.680	−151.141	−4.868	0.06	18.70
51.7	0.842	0.197	−17.967	43.408	2.220		
53.3	1.125	0.148	−16.987	30.403	1.663		
55.3	1.143	0.145	−16.244	32.126	1.636		
56.6	1.075	0.154	−15.696	36.948	1.739		
57.5	0.982	0.169	−15.254	43.306	1.904		
58.0	0.888	0.187	−14.882	50.421	2.105		
58.6	0.804	0.206	−14.547	58.311	2.325		
59.0	0.735	0.226	−14.242	66.083	2.546		
59.6	0.680	0.244	−13.988	73.637	2.749		

^a^The standard uncertainties for molality, temperature and pressure were *u* (*C*) = 0.001 mol m^−3^, *u* (*T*) = 0.5 K, and u(*P*) = 0.01 MPa respectively with level of confidence 0.95. The standard combined uncertainty for surface tension were about, *uc* (*γ*) = 0.01 mN m^−1^ (level of confidence 0.68), respectively.

Analysis of Table 3 revealed a direct correlation between the concentration of aqueous gabapentin solutions and the ${\Gamma _{\hbox{max} }}$ values. As the concentration of gabapentin increased, the ${\Gamma _{\hbox{max} }}$ values rose. Conversely, increasing the length of the alkyl chain in the ILs molecules led to a decrease in surface tension. These observations can be attributed to the interplay of hydrophobicity, hydrophilicity, and surface tension. At higher gabapentin concentrations, the molecule interacts with both water molecules and ILs, influencing their distribution at the air-water interface. In some instances, gabapentin may enhance the adsorption of ILs, increasing their surface concentration. However, longer alkyl chains in ILs can promote micelle formation in the bulk solution, reducing their adsorption at the surface. Moreover, stronger intermolecular forces within these micelles can hinder the detachment of ILs and their subsequent adsorption. The observed behavior of ILs in aqueous gabapentin solutions is a complex interplay of these factors. The decrease in ${\Gamma _{\hbox{max} }}$ values with increasing alkyl chain length suggests improved efficiency of ILs at the air-water interface, possibly due to more compact packing of molecules. The minimum occupied surface area per ILs molecule in varied aqueous gabapentin solution is defined as *A*_min_ and can be expressed as^46,48^:

3 $${A_{\hbox{min} }}=\frac{{{{10}^{20}}}}{{{N_A} \cdot {\Gamma _{\hbox{max} }}}}$$

Here, *N*_A_ is the Avogadro number. The related values of *A*_min_ have been presented in Table 3. The *A*_*min*_ values, as presented in Table 3, reveal a direct correlation with the alkyl chain length and an inverse relationship with the gabapentin concentration in aqueous solution. These findings can be attributed to the interplay between the ILs’ structural characteristics and their interfacial behavior at the air-liquid interface of the gabapentin solution. As the alkyl chain length increases, the hydrophobic effect becomes more pronounced, driving the ILs towards the interface and resulting in denser packing and a concomitant decrease in surface tension. The hydrophobic nature of the alkyl chain minimizes its contact with water, favoring interfacial localization. Gabapentin, depending on its concentration, can interact with both water molecules and the ILs at the interface. At higher concentrations, gabapentin may compete with the ILs for favorable interfacial positions, disrupting their packing efficiency and potentially increasing the minimum area occupied per molecule^49–52^. This disruption is likely responsible for the observed decrease in ${\Gamma _{\hbox{max} }}$ with an increase in the length alkyl chain.

Also, Table 3 present the thermodynamic parameters of micellization, including the Gibbs standard free energy of micellization ${\varDelta G}_{mic}^{0}$, the Gibbs free energy of surface at equilibrium $G_{{\hbox{min} }}^{s}$, and the Gibbs standard free energy of adsorption $\Delta G_{{ad}}^{0}$, for the investigated ILs in aqueous gabapentin solutions. These parameters can be calculated using the following expressions^53,54^:

4 $${\varDelta G}_{mic}^{0}=RT\text{ln}{X}_{cmc}$$

5 $${G}_{min}^{S}={A}_{min}{\gamma }_{CMC}{N}_{A}$$

6 $${\varDelta G}_{ad}^{0}={\varDelta G}_{mic}^{0}-\frac{{{\Pi }}}{{{\Gamma }}_{max}}$$

In the aforementioned equations, the symbol ${X_{cmc}}$ represents the molar fraction concentration of ILs in aqueous gabapentin solutions. A comprehensive analysis of Table 3 reveals that the micellization process is spontaneous, as evidenced by the consistently negative values of ${\varDelta G}_{mic}^{0}$. The ${\varDelta G}_{mic}^{0}$ values for the studied ILs exhibit a decreasing trend (becoming more negative) with increasing gabapentin concentration and alkyl chain length. Among the investigated ILs, [C_8_bet][Br] demonstrates the most negative ${\varDelta G}_{mic}^{0}$ values, indicating a strong thermodynamic preference for micellization.

The decrease in ${\varDelta G}_{mic}^{0}$ can be attributed to the rapid saturation of the surface medium by ionic liquids that feature surface activity properties from themselves (SAILs). This indicates that ILs molecules accumulate efficiently at the air-liquid interface, thereby creating a more favorable thermodynamic environment for micelle formation. The values of ${\varDelta G}_{ad}^{0}$, representing the Gibbs free energy of adsorption, are calculated using Eq. (6). A comparative analysis between ${\varDelta G}_{mic}^{0}$ and ${\varDelta G}_{ad}^{0}$reveals that ${\varDelta G}_{mic}^{0}$values are consistently more negative. This trend highlights a pronounced preference for ILs molecules to self-assemble into micelles in the bulk solution rather than remaining adsorbed at the surface. This thermodynamic inclination towards micellization underscores the significant role of intermolecular interactions in the bulk phase^53,54^. The parameter *Γ*_max_, which reflects the tendency of ILs to form a new surface at the solution interface, is found to be positive for all studied systems. This further corroborates the dominance of micellization and self-assembly processes, especially with increasing gabapentin concentration and alkyl chain length. These observations suggest that micelle formation is energetically more favorable than surface adsorption under the investigated conditions. The growing interest in studying ILs, ionic liquids with long alkyl chains, stems from their immense potential in applications such as nanotechnology and biomedicine^55^. The relationship between surfactant structure and solution properties plays a pivotal role in tailoring these materials for specific applications. Studies on 1-(1-alkyl)-3-methylimidazolium chlorides with alkyl chains containing 10, 12, and 14 carbons have demonstrated that the head-group structure, alkyl chain length, and interfacial interactions significantly influence micellar properties^55^. Comparison with other surfactant classes, such as 1-(1-alkyl)pyridinium chlorides and benzyl (2-acylaminoethyl)dimethylammonium chlorides, underscores the importance of hydrogen bonding, head-group acidity, and aromatic interactions in determining micellization behavior^55^. In addition to spherical micelles, ionic surfactants can also assemble into wormlike micelles (WLM) at higher concentrations, forming polymer-like supramolecular structures^56^. While the rheology of these aggregates is well studied, experimental thermodynamic insights at lower concentrations remain limited. For instance, investigations with tetradecyltrimethylammonium salicylate (TTASal) revealed that WLM formation is predominantly driven by surfactant ion-counterion interactions rather than concentration alone. These findings emphasize that enthalpy changes associated with aggregation processes critically dictate whether monomers aggregate into spherical micelles or WLM structures. This distinction provides valuable insight into the interplay between interfacial and bulk-phase thermodynamics in dictating the self-assembly behavior of ILs^56^.

### Electrical conductivity

**Fig. 3:**
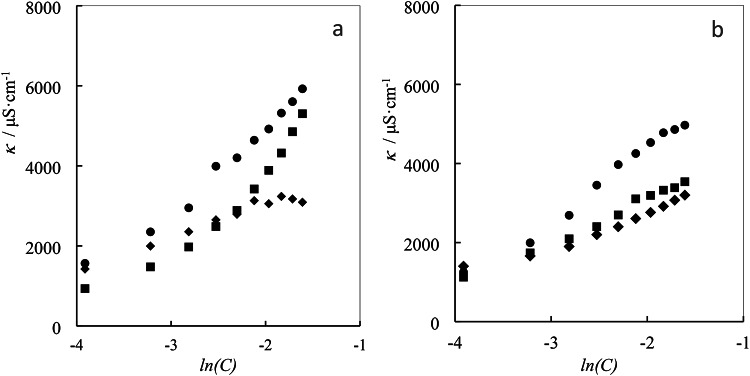
Specific conductance (*κ*) of betaine-based ILs: (**a**) ([C_4_bet][Br] (♦), [C_6_bet][Br] (■), and [C_8_bet][Br] (●)) in aqueous solutions and (**b**) ([C_4_bet][Br] (●), [C_6_bet][Br] (■), and [C_8_bet][Br] (♦)) in aqueous solutions of 0.01 mol kg^−1^gabapentin.

The molar conductivity values (*Λ*) the studied ionic liquids ([C_4_bet][Br], [C_6_bet][Br] and [C_8_bet][Br]) by concentrations variation of gabapentin in aqueous solutions at 298.15 K have been tabulated within Table 4 in and has been illustrated in Fig. 3.

**Table 4:** Specific conductance and molar conductivities of betaine-based IL in varied concentrations of aqueous gabapentin solutions as a function of ILs molarity (*c*) at 298.15 K.

m/mol·kg^−1^											
0			0.01			0.0299			0.0499		
*C*	*κ *	*Λ*	*C*	*κ *	*Λ*	*C*	*κ *	*Λ*	*C*	*κ *	*Λ*
(mol·kg^−1^)	(µS·cm^−1^)	(S·m^2^·mol^−1^)	(mol·kg^−1^)	(µS·cm^−1^)	(S·m^2^·mol^−1^)	(mol·kg^−1^)	(µS·cm^−1^)	(S·m^2^·mol^−1^)	(mol·kg^−1^)	(µS·cm^−1^)	(S·m^2^·mol^−1^)
[C_4_bet][Br]											
0.0200	1422	64.94	0.0201	1402	70.04	0.0200	970	48.58	0.0200	794	46.20
0.0400	1995	38.73	0.0401	1660	41.48	0.0400	1420	35.54	0.0401	1046	37.36
0.0600	2353	30.92	0.0601	1900	31.70	0.0601	1640	27.35	0.0600	1250	30.93
0.0800	2650	24.37	0.0801	2200	27.54	0.0799	1932	24.21	0.0797	1445	27.00
0.0999	2790	21.07	0.1000	2400	24.08	0.1004	2100	20.96	0.0999	1600	23.95
0.1201	3130	18.38	0.1202	2606	21.74	0.1200	2340	19.53	0.1199	1700	21.12
0.1400	3054	16.66	0.1403	2763	19.75	0.1401	2540	18.17	0.1411	1825	19.12
0.1599	3236	15.06	0.1603	2917	18.24	0.1599	2767	17.34	0.1589	1900	17.24
0.1799	3172	13.66	0.1803	3072	17.08	0.1800	3000	16.70	0.1790	1950	15.50
0.2007	3090	12.33	0.2006	3200	16.00	0.1982	3132	15.83	0.1995	1967	14.00
[C_6_bet][Br]											
0.0200	932.4	46.72	0.0200	1125	56.40	0.0200	700	35.11	0.0201	742.5	26.97
0.0399	1475	37.09	0.0400	1738	43.54	0.0399	1200	30.14	0.0400	1320	25.05
0.0599	1973	33.02	0.0598	2095	35.13	0.0599	1810	30.27	0.0599	1830	26.73
0.0796	2483	31.27	0.0798	2402	30.16	0.0800	2349	29.40	0.0794	2313	27.90
0.0999	2886	28.96	0.0998	2698	27.11	0.0997	2912	29.27	0.1001	2711	27.13
0.1196	3421	28.70	0.1199	3105	25.96	0.1200	3480	29.06	0.1199	3067	25.62
0.1380	3888	28.26	0.1397	3194	22.92	0.1399	3900	27.93	0.1392	3276	23.57
0.1602	4320	27.05	0.1576	3325	21.15	0.1595	4200	26.38	0.1599	3400	21.30
0.1801	4851	27.02	0.1782	3386	19.05	0.1795	4515	25.20	0.1797	3512	19.57
0.1994	5301	26.66	0.1996	3538	17.77	0.1986	4878	24.61	0.1968	3621	18.43
[C_8_bet][Br]											
0.0200	1564	78.60	0.0200	1252	62.85	0.0200	1204	60.39	0.0200	950	47.60
0.0400	2350	58.95	0.0400	1994	49.98	0.0398	1800	45.30	0.0400	1630	40.82
0.0599	2950	49.39	0.0599	2690	45.05	0.0599	2500	41.84	0.0598	2024	33.87
0.0797	3990	50.20	0.0797	3452	43.43	0.0801	3100	38.76	0.0799	2450	30.69
0.1000	4200	42.13	0.0997	3972	39.94	0.0999	3614	36.24	0.0999	2895	29.03
0.1199	4640	38.83	0.1199	4253	35.57	0.1198	3931	32.86	0.1198	3346	27.98
0.1394	4920	35.40	0.1400	4530	32.44	0.1398	4105	29.42	0.1401	3649	26.08
0.1599	5318	33.36	0.1600	4778	29.95	0.1600	4250	26.61	0.1600	3865	24.20
0.1801	5603	31.20	0.1800	4860	27.07	0.1783	4360	24.50	0.1799	3970	22.10
0.2000	5924	30.09	0.1995	4970	24.98	0.1997	4468	22.41	0.1994	4000	20.09

^a^ The standard uncertainties for molality and temperature were *u* (*C*) = 0.001 mol m^−3^ and *u* (*T*) = 0.5 K, respectively with level of confidence 0.95. The standard combined uncertainty for conductance and molar conductivity were about, *uc* (*κ*) = 0.5 µS·cm^−1^ and *uc*(*Λ*) = 0.7 S·m^2^·mol^−1^ (level of confidence 0.68), respectively.

**Fig. 4:**
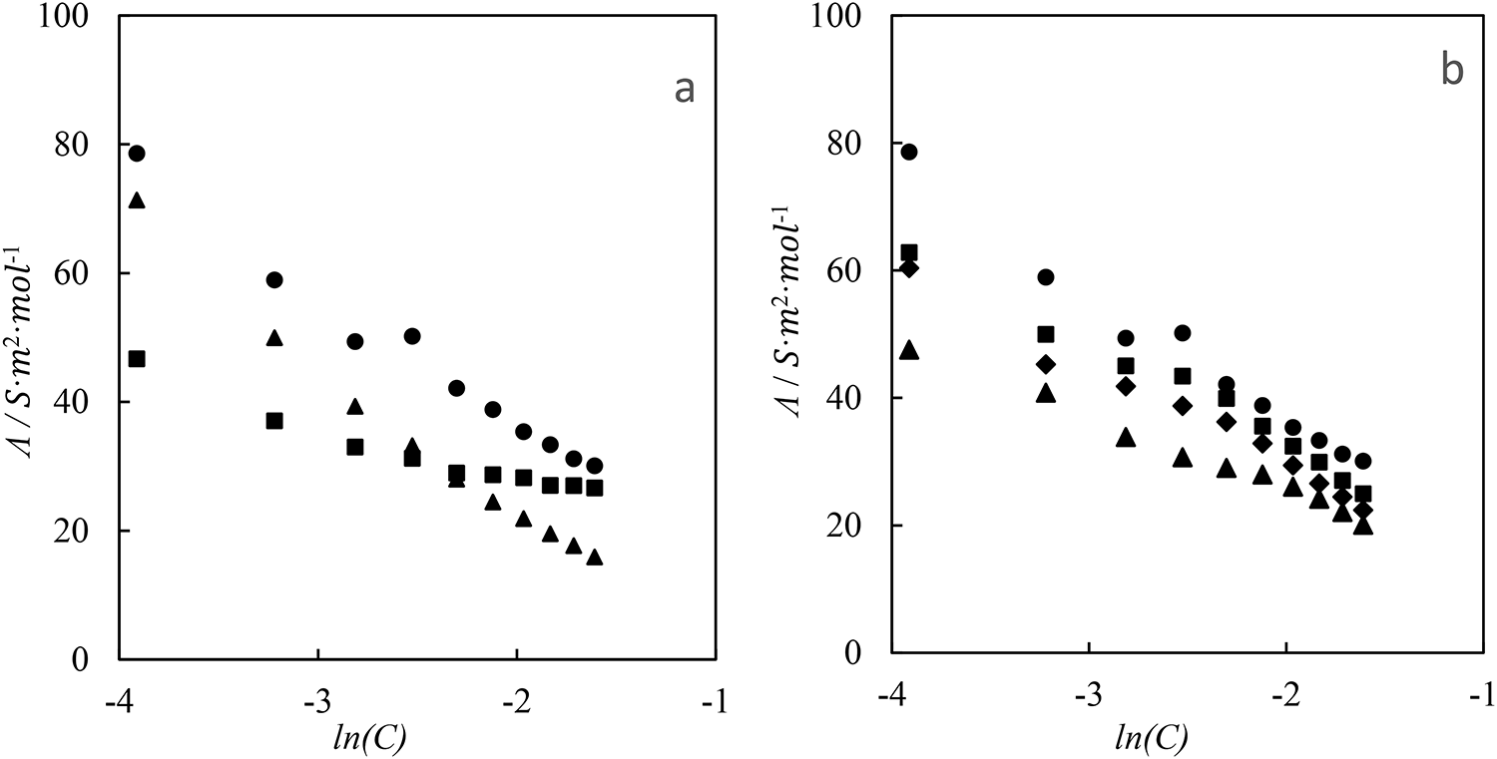
Molar conductivities (*Λ*) of betaine-based ILs: (**a**) ([C_4_bet][Br] (▲), [C_6_bet][Br] (■), and [C_8_bet][Br] (●)) in aqueous solutions, and (**b**) in the presence of (●) 0.00 mol kg^−1^, (■) 0.01 mol kg^−1^_,_ (♦) 0.03 mol kg^−1^, and (▲) 0.05 mol kg^−1^ gabapentin in aqueous solution of [C_8_bet][Br] at 298.15 K.

Figure 4 illustrates the dependence of *Λ* on ILs concentration in different aqueous gabapentin concentrations.

The *Λ* values exhibit a clear decrease as the concentrations of ILs increase. Additionally, these values also decrease with an increase in the content of gabapentin. The following formula were utilized to evaluate the experimental data using the low concentration Chemical Model (lcCM)^57,58^:

7 $$\Lambda = \alpha \left[\Lambda_0-S(c\alpha)^{1/2} + EC\alpha \ln (c\alpha) + J_{1} c\alpha + J_{2} (c\alpha)^{3/2} \right]$$

8 $$K_{A} = \frac{1-\alpha}{\alpha^{2}c\gamma_{\pm}^{2}}$$

9 $$\ln \gamma_{\pm} = - \frac{kq}{1+kR}$$

10 $$k^{2}=\frac{16{,}000N_{A}z^{2}e^{2}\alpha c}{\varepsilon_{0}\varepsilon k_{B} T}$$

11 $$q = \frac{z^{2}e^{2}}{8\pi \varepsilon_{0} \varepsilon k_{B} T}$$

where *Λ*_0_ represents the limiting molar conductivity, ($1- \alpha$) is the percentage of oppositely charged ions functioning as ion pairs, *R* is the distance parameter, and $\gamma_{\pm}$is the corresponding mean activity gabapentin of free ions. The numbers needed for the coefficients *E*, *J*_1_ and *J*_2_ computations were obtained from Barthel and co-workers. In the above cited equations, the *c*, represent the molar of ILs that has been computed from following expression:

12 $$c=m \cdot \rho$$

The symbol *m* represents the molality of the prepared solutions, and the *ρ* denotes the density of the aqueous gabapentin solutions. The densities of the solutions with molalities of 0.0000, 0.0100, 0.0300, and 0.0500 mol·kg^−1^ are 997.042 kg·m^2^, 997.34 kg·m^2^, 998.13 kg·m^2^, and 998.60 kg·m^2^, respectively. The remaining parameters in the aforementioned equations have their usual meanings.

By employing non-linear least-squares fitting on the molar conductivity data, we can derive the ion-association constant (*K*_*A*_) the limiting molar conductivity (*Λ*_0_) and the distance parameter (*R*). Table 5 presents the values of *K*_*A*_, *Λ*_0_, and *R* for both binary and ternary systems.

**Table 5:** The association constants (*K*_A_), limiting molar conductivities (*Λ*_0_), the distance of closest approach of ions (*R*), and standard deviations (*S*_dev_) of betaine-based ILs in ternary aqueous solutions at 298.15 K.

*m* (mol·kg^−1^)	*K*_A_ (dm^3^·mol^−1^)	*Λ*_0_ (S·m^2^·mol^−1^)	10^10^ *R* (mol·kg^−1^)	S_dev_
[C_4_bet][Br] in aqueous solution of gabapentin				
0.0000	30,314	2030	60.08	1.14
0.0100	1963	695.7436	57.93	0.75
0.0299	2799	427.3586	45.46	0.58
0.0499	3000	386.1030	52.52	0.38
[C_6_bet][Br] in aqueous solution of gabapentin				
0.0000	1094.6	212.8081	0.487	0.41
0.0100	289.3459	135.7082	32.77	0.32
0.0299	9.2934	36.997	0.915	0.56
0.0499	25.2102	49.7324	2.743	0.87
[C_8_bet][Br] in aqueous solution of gabapentin				
0.0000	917.5686	342.0580	8.93	0.92
0.0100	161.3077	138.4277	4.573	0.98
0.0299	361.8653	183.6283	1.072	0.88
0.0499	86.5474	93.4026	4.316	1.32

^a^ The standard uncertainties for molality and temperature were *u* (*C*) = 0.001 mol m^−3^ and *u* (*T*) = 0.5 K, respectively with level of confidence 0.95. The standard combined uncertainty for conductance and molar conductivity were about, *uc* (*κ*) = 0.5 µS·cm^−1^ and *uc*(*Λ*) = 0.7 S·m^2^·mol^−1^ (level of confidence 0.68), respectively.

As shown in the Table 5, the limiting molar conductivity *Λ*_0_ values decrease with increasing gabapentin concentration. This phenomenon can be attributed to two competing effects: (a) Increased ion-ILs interactions: As gabapentin concentration rises, the interactions between ILs and gabapentin become stronger. This can lead to larger solvated ion radii, potentially increasing their mobility. (b) Increased solution viscosity: A higher gabapentin concentration can also increase the viscosity of the solution. This, in turn, can reduce the mobility of ions due to increased resistance to movement.

However, for the binary ILs systems, the limiting molar conductivity *Λ*_0_ values increase with increasing alkyl chain length. The observed order of *Λ*_0_ values for the studied systems is [C_4_bet][Br] < [C_6_bet][Br] < [C_8_bet][Br], suggesting that [C_4_bet][Br] exhibits the most intense interactions due to its lower *Λ*_0_ value. This reduced mobility of [C_4_bet][Br] may be attributed to the shorter alkyl chain length, which could lead to more compact and less mobile ion-ILs complexes.

### Critical micelle concentration (CMC)

SAILs, when introduced to a solution’s surface, exhibit a tendency to aggregate in a manner where their long alkyl chains orient towards the air-water interface while their hydrophilic head groups penetrate the water surface. This process continues until the surface becomes saturated with SAILs molecules. Beyond this point, excess SAILs enter the bulk phase of the solution.

Within the bulk phase, the hydrophobic tails of SAILs aggregate to minimize contact with water, while the hydrophilic heads remain oriented towards the aqueous environment. This aggregation phenomenon leads to the formation of micelles at a specific concentration known as the Critical Micelle Concentration (CMC). Below the CMC, SAILs molecules are dispersed individually throughout the solution. At and above the CMC, SAILs molecules self-assemble into micelles, with the hydrophobic tails forming the micelle core and the hydrophilic heads facing outwards to interact with the water. The structure of SAILs significantly influences their CMC point, with longer alkyl chains generally resulting in lower CMC values. This is because longer alkyl chains enhance the hydrophobicity, promoting micelle formation at lower concentrations. The CMC of three SAILs ([C_4_bet][Br], [C_6_bet][Br], and [C_8_bet][Br]) in the presence of varying concentrations of aqueous gabapentin solutions at 298.15 K. Static surface tension measurements using the Wilhelmy plate method and electrical conductivity measurements were employed as reliable and accurate techniques to determine the CMC. The CMC in both methods was obtained by extrapolating the inflection point in the specific conductance and surface tension plots against the measured solution molality. Gabapentin, when added to the bulk phase of the solution, occupies a relatively large volume due to its tendency to dissolve in water in a molecular-like manner. This behavior is supported by its specific pH range of 6.5-8 and its non-conductive nature. Understanding this phenomenon holds significance for researchers in both industrial and pharmaceutical fields. Table 3 tabulates a decrease in the CMC of SAILs with increasing gabapentin concentration which can be attributed to the enhanced hydrophobicity associated with longer alkyl chains. This hydrophobicity promotes micelle formation at lower concentrations.

The addition of gabapentin to the aqueous solution disrupts the favorable interactions between water and the hydrophilic head groups of SAILs, creating a less favorable environment for the hydrophobic tails to reside in the bulk phase. Consequently, SAILs preferentially aggregate with each other to minimize this unfavorable interaction. This aggregation leads to a reduction in the concentration of free SAILs molecules in the solution, effectively lowering the overall CMC. The variation in electrical conductivity and surface tension with the concentration of the studied SAILs is also observed to decrease with increasing gabapentin concentration^59^. This trend further supports the notion that gabapentin molecules accumulate in the solution, interfering with the interactions between SAILs and water. The observed increase in the specific conductivity of SAILs with increasing alkyl chain length can be attributed to several factors. Longer alkyl chains provide greater separation between the charged head groups, facilitating ionic dissociation and increasing the overall ionic character of the SAILs. As alkyl chain length increases, the hydrophobicity of the SAILs also increases, making it energetically more favorable for individual SAILs molecules to remain dispersed in the solution rather than forming micelles, especially at lower concentrations. The combined effects of increased ionic character and reduced micelle formation result in enhanced ion mobility. With more ions present in the solution and fewer trapped within micelles, the ions can move more freely, contributing to higher conductivity. The hydrophobic interactions between the alkyl chains of SAILs can also influence conductivity. Longer alkyl chains may exhibit stronger hydrophobic interactions, leading to the formation of more compact micelles or aggregates. These compact structures can potentially restrict the mobility of ions within the aggregates, potentially affecting conductivity^60–62^. As depicted in Fig. 2, an increase in specific conductivity of SAILs with increasing alkyl chain length is primarily driven by the enhanced ionic character, reduced micelle formation, and increased ion mobility. While hydrophobic interactions between alkyl chains can also play a role, their influence on conductivity is likely less significant compared to the aforementioned factors.

### Molecular fingerprints of the ILs and gabapentin

The DFT-COSMO calculation for hydration cavity properties of the ILs and gabapentin are given in Table 6.

**Table 6:** The surface area and total volume of cavity, dielectric (solvation) energy, HOMO and LUMO values and energies obtained from COSMO and Dmol3 calculations.

Material	*A* (A^2^)	*V* (A^3^)	Dielectric (solvation) energy (kcal·mol^−1^)	HOMO	LUMO	E_HOMO_ ev	E_LUMO_ ev
Gabapentin	198.232	202.454	−26.60	47	48	−5.531	−0.362
[C_4_bet][Br]	276.193	267.429	−96.00	66	67	−4.886	−1.707
[C_6_bet][Br]	316.051	304.866	−103.10	74	75	−4.776	−1.658
[C_8_bet][Br]	363.545	345.454	−102.05	82	83	−4.954	−1.624

**Fig. 5:**
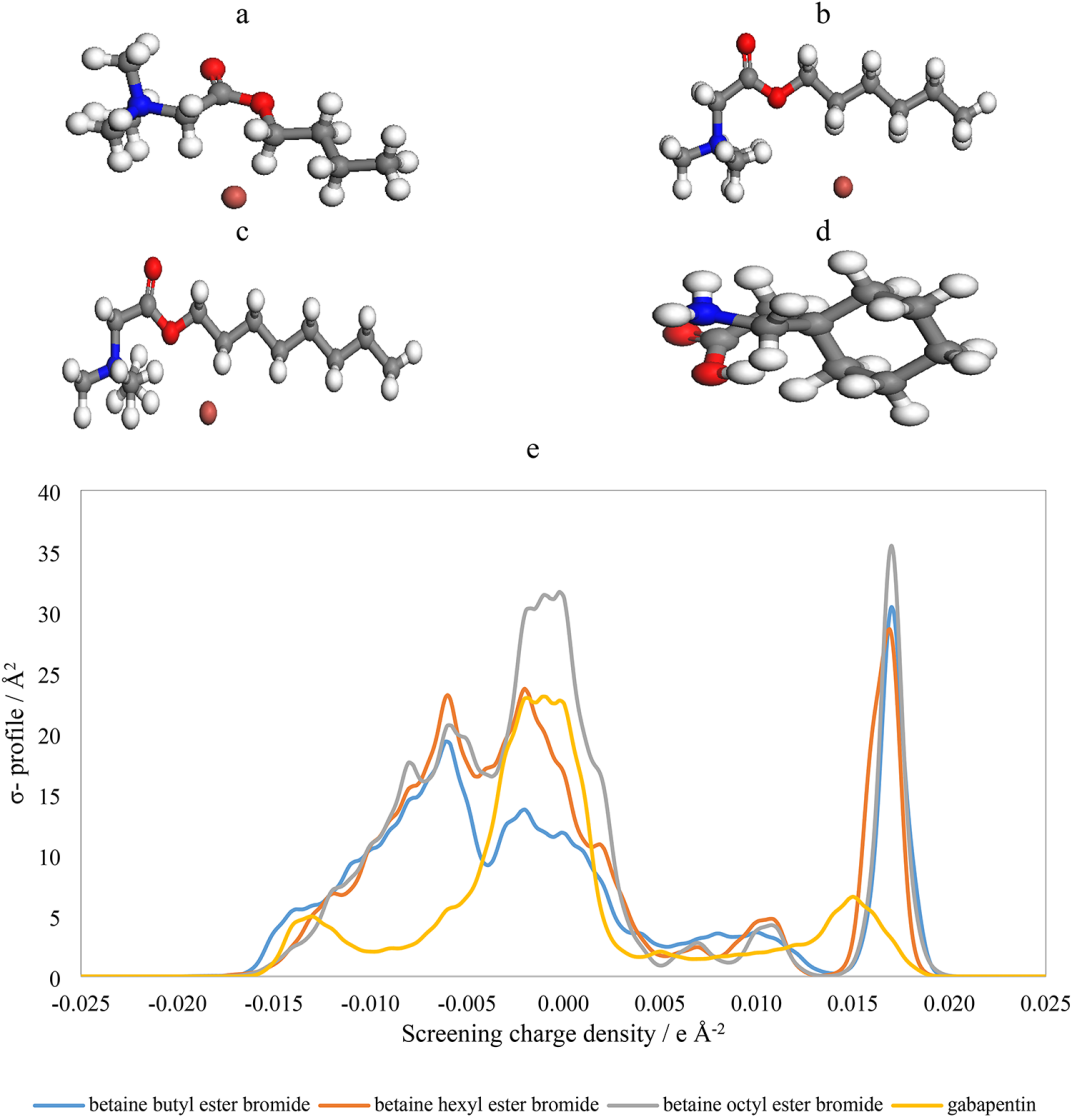
Optimized molecular structure of (**a**) [C_4_bet][Br], (**b**) [C_6_bet][Br], (**c**) [C_8_bet][Br], (**d**) Gabapentin, and (**e**) *σ*-profile plots from Dmol3 and COSMO result.

The results for gabapentin and the betaine-based ionic liquids ([C_4_bet][Br], [C_6_bet][Br], and [C_8_bet][Br]) illustrate key relationships between cavity volume, surface area, and dielectric energy in the context of micellization behavior. The optimized structures and the *σ*- profiles are given in Fig. 5.

Gabapentin has a relatively small cavity volume and surface area, resulting in a less negative dielectric energy of -26.60 kcal/mol, indicating less favorable solvation and a diminished ability for effective micellization. In contrast, [C_4_bet][Br] exhibits increased cavity volume (267.429 Å^2^) and surface area (276.193 Å^2^), with a more negative dielectric energy of -96.00 kcal/mol, suggesting stronger solvation and better potential for micellization. This trend continues with [C_6_bet][Br], which has even larger cavity volume (304.866 Å^2^) and surface area (316.051 Å^2^) and a dielectric energy of -103.10 kcal/mol, indicating improved solvation properties that facilitate micelle stability. Although [C_8_bet][Br] has the largest cavity volume (345.454 Å^2^) and surface area (363.545 Å^2^), its dielectric energy of -102.05 kcal/mol is slightly less negative than that of [C_6_bet][Br]. Nevertheless, its size still suggests strong micellization potential. Overall, the increasing cavity volume and surface area from gabapentin to the betaine ionic liquids enhance hydrophobic interactions and solvation, thereby facilitating micellization. The trend of more negative dielectric energies further supports this by indicating better solvation and increased stability of micelles, particularly for [C_4_bet][Br] and [C_6_bet][Br].

The *σ*- profiles show that the [C_6_bet][Br] and gabapentin near the neutral screening charge density are more compatible that is confirmation to the results that has evaluated in the conductometery and association constant results that show the lowest among the studied ILs. It happenes to existing of the gabapentin in the aquesous solution of the ILs that has the similar charge density and different structure led to dispersssion of the IL more than the other studied ILs. The lower association constants, especially for [C_6_bet][Br], highlight the presence of weaker interactions, which can be attributed to the molecular dispersion forces rather than strong ionic interactions. The structural difference between gabapentin and [C_6_bet][Br], despite having similar charge densities, seems to promote the dispersion of [C_6_bet][Br] more than the other studied ILs.

In aqueous solutions, the presence of gabapentin leads to different behavior for the ILs, with [C_6_bet][Br] dispersing more readily. This is likely due to gabapentin’s similar charge density to [C_6_bet][Br], although their structures are different. The interaction between [C_6_bet][Br] and gabapentin in the solution appears to be weaker, which facilitates better dispersion compared to other ILs like [C_4_bet][Br] and [C_8_bet][Br]. As a result, [C_6_bet][Br] shows less interaction with gabapentin, leading to a more stable and dispersed system in aqueous media. The surface tension values measured in the study also reflect this trend. For example, the surface tension of [C_4_bet][Br] in water decreases as the concentration increases, while in gabapentin solutions, surface tension values shift more dramatically with increasing concentrations. This suggests that the interaction between the ILs and gabapentin is concentration-dependent, with higher concentrations of gabapentin resulting in more significant changes in surface properties. For [C_6_bet][Br], the lower surface tension values in these solutions further support the idea of weaker interactions and greater dispersion.

The free energy of solvation provides additional insight into these interactions. For [C_6_bet][Br], the negative free energy of solvation in solutions with gabapentin indicates favorable solute-solvent interactions, though extremely negative values at higher concentrations of gabapentin suggest more complex interactions. Despite this, dispersion still plays a significant role, as reflected in the σ-profile data and the lower association constant values for [C_6_bet][Br]. In comparing the ILs, it is clear that [C_6_bet][Br] demonstrates the weakest interactions with gabapentin, evidenced by its lower association constants and behavior in solution. On the other hand, [C_4_bet][Br] and [C_8_bet][Br] show stronger interactions with gabapentin, forming more structured systems. For example, [C_8_bet][Br], with its longer hydrophobic chain, exhibits significant energy fluctuations and changes in surface tension, suggesting a more organized interaction with the aqueous phase compared to shorter-chain ILs like [C_6_bet][Br].

## Conclusion

This study thoroughly examined the interfacial, micellization, and electrical conductivity properties of betaine-based ionic liquids that featured surface-activity properties from themselves, [C_4_bet][Br], [C_6_bet][Br], and [C_8_bet][Br], in aqueous the drug gabapentin solutions at varying concentrations (0.0000, 0.0100, 0.0300, and 0.0500) mol kg⁻¹ at 298.15 K. The surface tension of the solutions decreased with increasing gabapentin concentration and alkyl chain length, emphasizing the influence of hydrophobic and hydrophilic interactions. Interfacial parameters, including surface pressure (*Π*), critical micelle concentration (CMC), Gibbs maximum excess surface concentration (*Γ*_*max*_), and minimum surface area per molecule (*A*_*min*_), indicated that longer alkyl chains enhance both interfacial efficiency and micellization tendencies. At higher gabapentin concentrations, the compact molecular packing of ILs at the interface was disrupted, leading to increased *A*_*min*_ values. Thermodynamic analysis revealed that the Gibbs free energy of micellization (*ΔG°*_*mic*_) and adsorption (*ΔG°*_*ad*_) were negative, confirming the spontaneous nature of micelle formation. The ILs with longer alkyl chains, particularly [C_8_bet][Br], showed more negative *ΔG°*_*mic*_ values, reflecting a thermodynamically favorable micellization process driven by strong hydrophobic interactions. A preference for micelle formation in the bulk phase over surface adsorption highlighted the importance of intermolecular interactions in stabilizing micelles. Electrical conductivity measurements demonstrated that the limiting molar conductivity (*Λ*_0_) decreased with increasing gabapentin concentration, attributable to higher solution viscosity and gabapentin-ILs interactions, but increased with longer alkyl chains due to enhanced ionic character. Ion association constants (*K*_*A*_) and distance parameters (*R*) confirm the formation of stable solvated ion pairs, which were more pronounced at higher gabapentin concentrations and with shorter alkyl chains. Molecular insights obtained via DFT-COSMO revealed that the cavity volume and surface area of the ionic liquids increased with alkyl chain length, promoting stronger hydrophobic interactions and facilitating micellization. Gabapentin, with its smaller cavity volume and less negative dielectric energy compared to the ILs, exhibited limited micellization potential but significantly influenced ILs solvation and interfacial behavior. In summary, the findings highlight the intricate interplay of structural characteristics, hydrophobicity, and solvation effects in determining the interfacial and bulk-phase behavior of betaine-based ILs in the presence of gabapentin. 

## Electronic supplementary material

Below is the link to the electronic supplementary material.

Supplementary Material 1

## Data Availability

All data generated or analyzed during this study are included in this article.
