# Supplementary material for: Effect of gabapentin on solution surface properties and micellization behavior of betaine-based surfactant ionic liquids
Source: Sci Rep. 2025 Jan 2;15:28. doi: 10.1038/s41598-024-83777-7 (PMC11695968; doi:10.1038/s41598-024-83777-7)
Supplement: Supplementary file 1 — Supplementary Material 1 [file 41598_2024_83777_MOESM1_ESM.docx]

Supporting Information

**Effect of Gabapentin on Solution Surface Properties and Micellization Behavior of Betaine-Based Surfactant Ionic Liquids**

**Shima Ghasemzadeh^1^, Mohammad Bagheri^1^, Hemayat Shekaari^1^*, Behrang Golmohammadi^1^**

^1^Department of Physical Chemistry, University of Tabriz, Tabriz, Iran

*Corresponding author.

Tel: +98 4133393094. Fax: 98 4133340191.

E-mail addresses: [hemayatt@yahoo.com](mailto:hemayatt@yahoo.com).


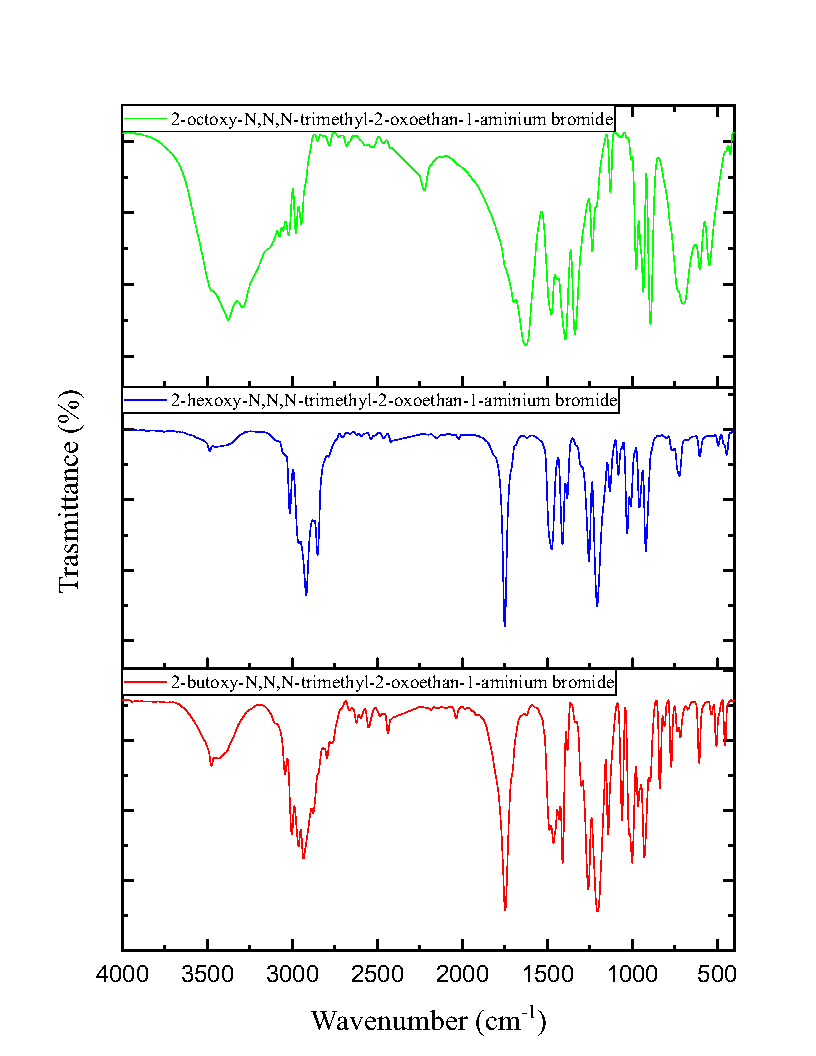


**Figure S1.** The FT-IR spectra of the synthesized ionic liquids.


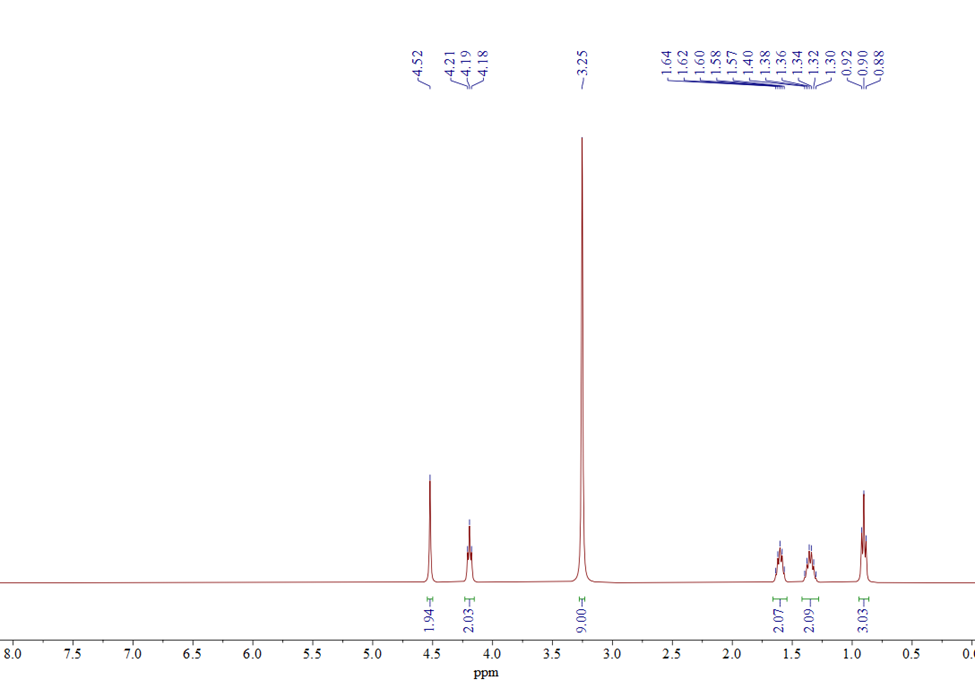

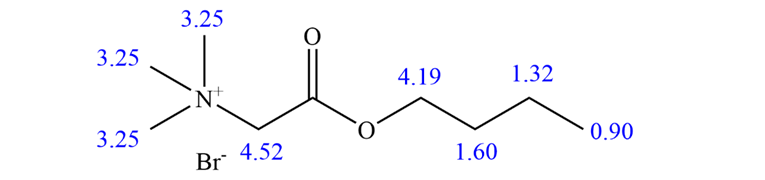


**Figure S2.** The 1HNMR spectra of 2-butoxy-N,N,N-trimethyl-2-oxoethan-1-aminium bromide.

^1^H NMR (400 MHz, DMSO) δ 4.52 (s, 2H), 4.19 (t, *J* = 6.6 Hz, 2H), 3.25 (s, 9H), 1.60 (p, *J* = 7.0 Hz, 2H), 1.35 (h, *J* = 7.4 Hz, 2H), 0.90 (t, *J* = 7.4 Hz, 3H).


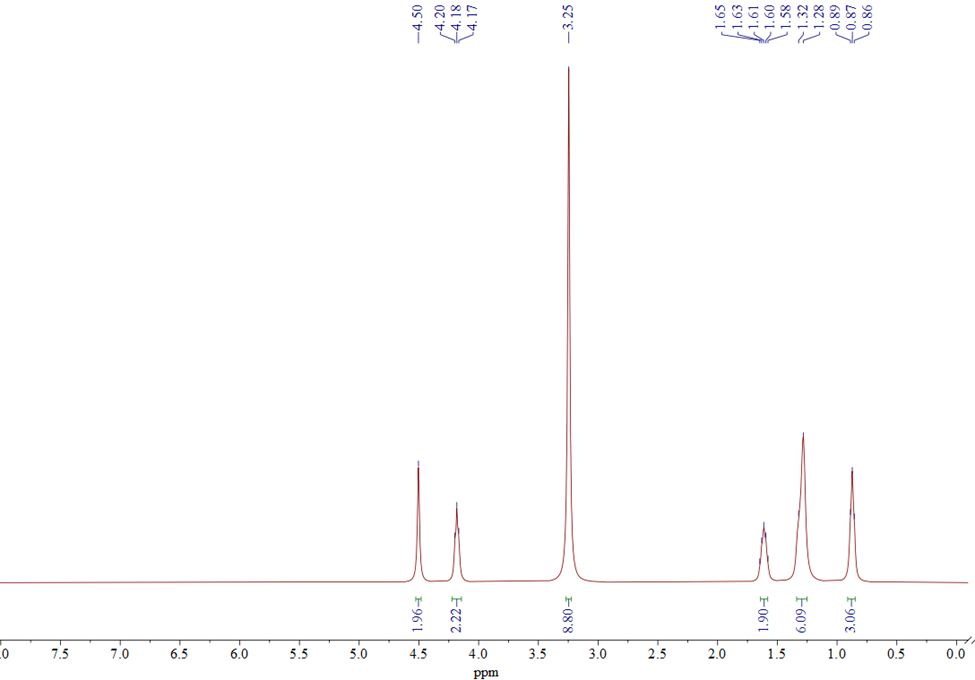

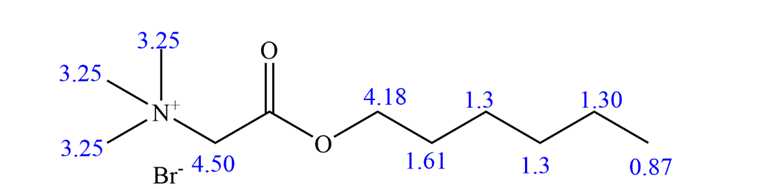


**Figure S3.** The 1HNMR spectra of 2-hexoxy-N,N,N-trimethyl-2-oxoethan-1-aminium bromide.

^1^H NMR (400 MHz, DMSO) δ 4.50 (s, 2H), 4.18 (t, *J* = 6.5 Hz, 2H), 3.25 (s, 9H), 1.61 (p, *J* = 6.7 Hz, 2H), 1.32 (s, 2H), 1.28 (s, 4H), 0.88 (d, *J* = 6.1 Hz, 3H).


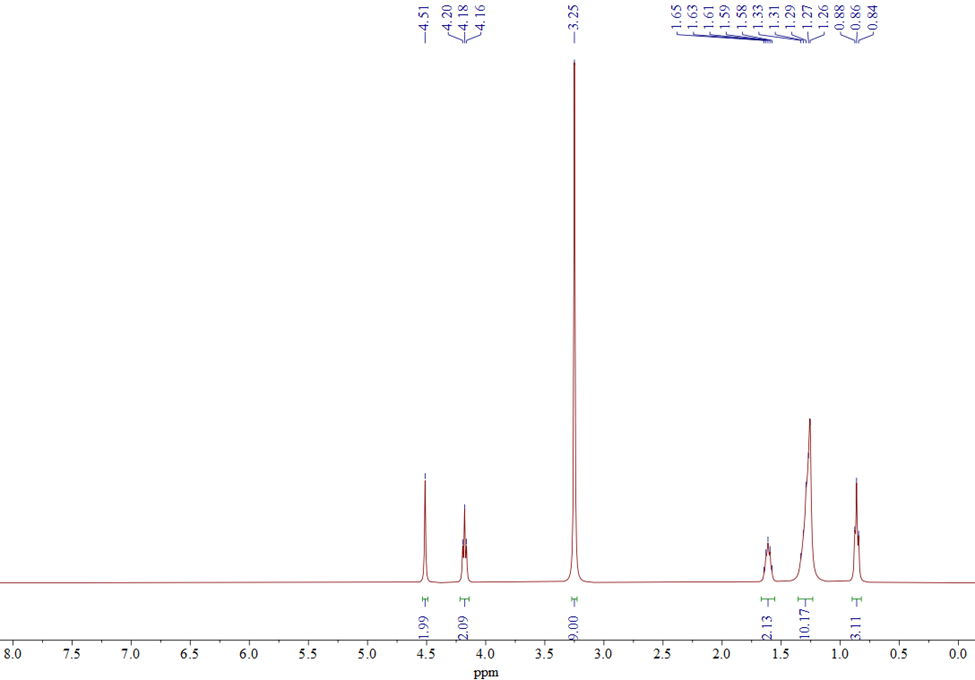

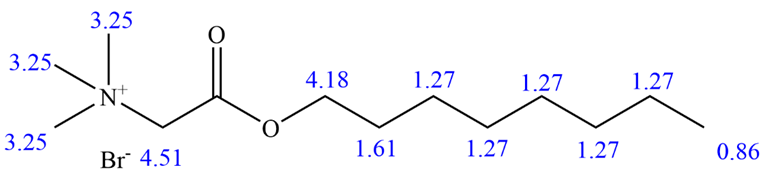


**Figure S4.** The 1HNMR spectra of 2-octoxy-N,N,N-trimethyl-2-oxoethan-1-aminium bromide.

^1^H NMR (400 MHz, DMSO) δ 4.51 (s, 2H), 4.18 (t, *J* = 6.6 Hz, 2H), 3.25 (s, 9H), 1.61 (p, *J* = 6.7 Hz, 2H), 1.28 (dd, *J* = 15.2, 7.3 Hz, 10H), 0.86 (t, *J* = 6.5 Hz, 3H).
